# Cognitive and Affective Empathy in Eating Disorders: A Systematic Review and Meta-Analysis

**DOI:** 10.3389/fpsyt.2019.00102

**Published:** 2019-03-04

**Authors:** Jess Kerr-Gaffney, Amy Harrison, Kate Tchanturia

**Affiliations:** ^1^Department of Psychological Medicine, Institute of Psychiatry, Psychology, and Neuroscience, King's College London, London, United Kingdom; ^2^Department of Psychology and Human Development, University College London, London, United Kingdom; ^3^Psychological Medicine Clinical Academic Group, South London and Maudsley NHS Trust, National Eating Disorders Service, London, United Kingdom; ^4^Department of Psychology, Ilia State University, Tbilisi, Georgia

**Keywords:** empathy, eating disorders, anorexia nervosa, autism, self-report, insight

## Abstract

**Background:** Recent models of eating disorders (EDs) have proposed social and emotional difficulties as key factors in the development and maintenance of the illness. While a number of studies have demonstrated difficulties in theory of mind and emotion recognition, little is known about empathic abilities in those with EDs. Further, few studies have examined the cognitive-affective empathy profile in EDs. The aim of this systematic review and meta-analysis was to provide a synthesis of empathy studies in EDs, and examine whether those with EDs differ from healthy controls (HC) on self-reported total, cognitive, and affective empathy.

**Methods:** Electronic databases were systematically searched for studies using self-report measures of empathy in ED populations. In total, 17 studies were identified, 14 of which could be included in the total empathy meta-analysis. Eight of the 14 studies were included in the cognitive and affective empathy meta-analyses.

**Results:** Meta-analyses showed that while total empathy and affective empathy scores did not differ between those with anorexia nervosa (AN) and HC, those with AN had significantly lower cognitive empathy scores compared to HCs (small effect size). Meta-analyses of Interpersonal Reactivity Index sub-scores revealed that AN had significantly lower Fantasy scores than HC (small effect size), indicating that those with AN have more difficulty in identifying themselves with fictional characters. Only 3 studies examined empathy in those with bulimia nervosa (BN) or binge eating disorder (BED).

**Conclusions:** The lowered cognitive empathy and intact affective empathy profile found in AN is similar to that found in other psychiatric and neurodevelopmental conditions, such as autism spectrum disorder (ASD). These findings add to the literature characterizing the socio-emotional phenotype in EDs. Future research should examine the influence of comorbid psychopathology on empathy in EDs.

## Introduction

### Rationale

Empathy refers to our ability to understand and identify the mental states of others, as well as our ability to share the feelings of others (1). It is considered a key component of social cognition, cooperation, and prosocial behavior, as it allows us to make sense of and respond appropriately to other people's behavior (2). Empathy can be separated into two major facets. Cognitive empathy refers to the ability to recognize and understand another's mental state (part of theory of mind (ToM) or mentalising) while affective empathy is the ability to share the feelings of others, without any direct emotional stimulation to oneself (3). As an illustrative example, sharing the excitement of a close friend's job offer is fundamentally different from understanding that your friend must be having thoughts and feelings, and what these feelings might be. These two aspects of empathy rely on different brain structures and take different developmental pathways, with affective empathy developing much earlier than cognitive empathy (1).

Differences in empathic abilities have been observed in a number of psychiatric disorders including schizophrenia (4, 5), autism spectrum disorder [ASD; (6, 7)], borderline personality disorder [BPD; (8)], and depression (9). Importantly, far from there being a universal deficit in empathic abilities, research in these psychiatric disorders shows that there is often a difficulty in a specific aspect of empathy, while other empathic abilities remain intact. For example, it has been found that those with ASD have problems with cognitive empathy, but do not differ from neurotypical controls in affective empathy (10). Reduced attention to informative social information may provide one explanation for the problems in cognitive empathy seen in those with ASD. For example, it is reported that individuals with ASD pay less attention to faces, and especially eyes (11), and this is associated with poorer emotion recognition and ToM ability (12–14), as well as lower social competence (15). Similarly, while healthy controls (HCs) show significantly higher levels of cognitive empathy compared to affective empathy, those with BPD show significantly poorer cognitive empathy than HCs, and slightly increased levels of affective empathy (16). In bipolar disorder (BD), this cognitive/affective empathy distinction is further complicated by clinical state. In both manic and depressive phases of illness, there is an impairment in cognitive empathy compared to HCs. However, during the manic phase, affective empathy is significantly higher than in HCs and patients in the depression phase of BD, who did not differ from one another (17). Increased affective empathy in BPD and BD may be related to disturbances in emotion inhibition.

Recent models of eating disorders (EDs) have put forward social and emotional difficulties as key factors in the development and maintenance of the illness (18, 19). However, relatively little is known about the specific empathy profile in those with EDs. Based on longitudinal research in a community sample from Sweden, Gillberg et al. published a number of papers reporting a subgroup of AN patients with “empathy disorders”—those that had severe problems in social understanding and communication, consistent with ASD (20). Poorer outcomes were found in this group (21, 22). Since then, a growing body of evidence has documented overlap between symptoms in ASD and AN. For example, both groups show high levels of social anxiety (23, 24) and alexithymia (25, 26), differences in social attention (11, 27, 28), and poorer emotion recognition (29, 30) and ToM ability (31, 32). Reduced social networks have been documented in AN and bulimia nervosa (BN) (33, 34), as well as difficulties in understanding the concept of friendship (35). It is possible that reduced empathic abilities, along with communication difficulties, may contribute to the diminished social networks and isolation that characterize EDs. Given that interpersonal difficulties are associated with more severe ED psychopathology (36, 37), understanding mechanisms that may contribute to these problems may be helpful in improving outcomes in those with these EDs.

### Objectives

The aim of this systematic review and meta-analysis is to provide a synthesis of empathy research in EDs. Previous reviews on social processes in EDs have ascribed relatively little attention to the topic, and focus on emotion recognition rather than other aspects of empathy such as affect sharing [e.g., (31)]. In addition, new studies have been published in the intervening years. An additional aim is to examine potential differences between those with EDs and HCs in the specific types of empathy (self-reported cognitive and affective empathy), to permit better comparisons with other psychiatric populations. Self-reported empathy measures will be the focus of this review, in order to elicit patients' views and self-assessment of their skills.

### Research Questions

The research questions are as follows: (1) do levels of self-reported empathy differ in those with EDs compared to HCs? (2) do levels of cognitive and affective empathy differ between EDs and HCs? (3) are empathy levels associated with any psychopathological or clinical variables?

## Methods

### Systematic Review Protocol

The review and meta-analysis was conducted using the Preferred Reporting Items for Systematic Reviews and Meta-Analyses (PRISMA) statement (38).

### Eligibility Criteria

Studies using a self-report measure of empathy were included. Inclusion criteria were: (1) means and standard deviations reported for empathy scores in at least one clinical ED group and a HC group (2) the clinical ED group met criteria for any ED diagnosis, according to DSM or ICD criteria (3) full article available in English (4) published in a peer reviewed journal. Articles that examined disordered eating samples rather than a clinical ED were not included.

### Data Sources and Search Strategy

The electronic databases SCOPUS, Web of Science, PsycInfo, and PubMed were searched for papers up to September 2018. The following search terms were used: anorexia nervosa OR bulimia nervosa OR eating disorder AND empathy OR emotional empathy OR empathic concern OR interpersonal reactivity. No other search limits were applied, with the exception of Web of Science, where results were filtered by the ED term for relevance. Reference lists were also searched for relevant papers.

### Study Selection

**Figure 1:**
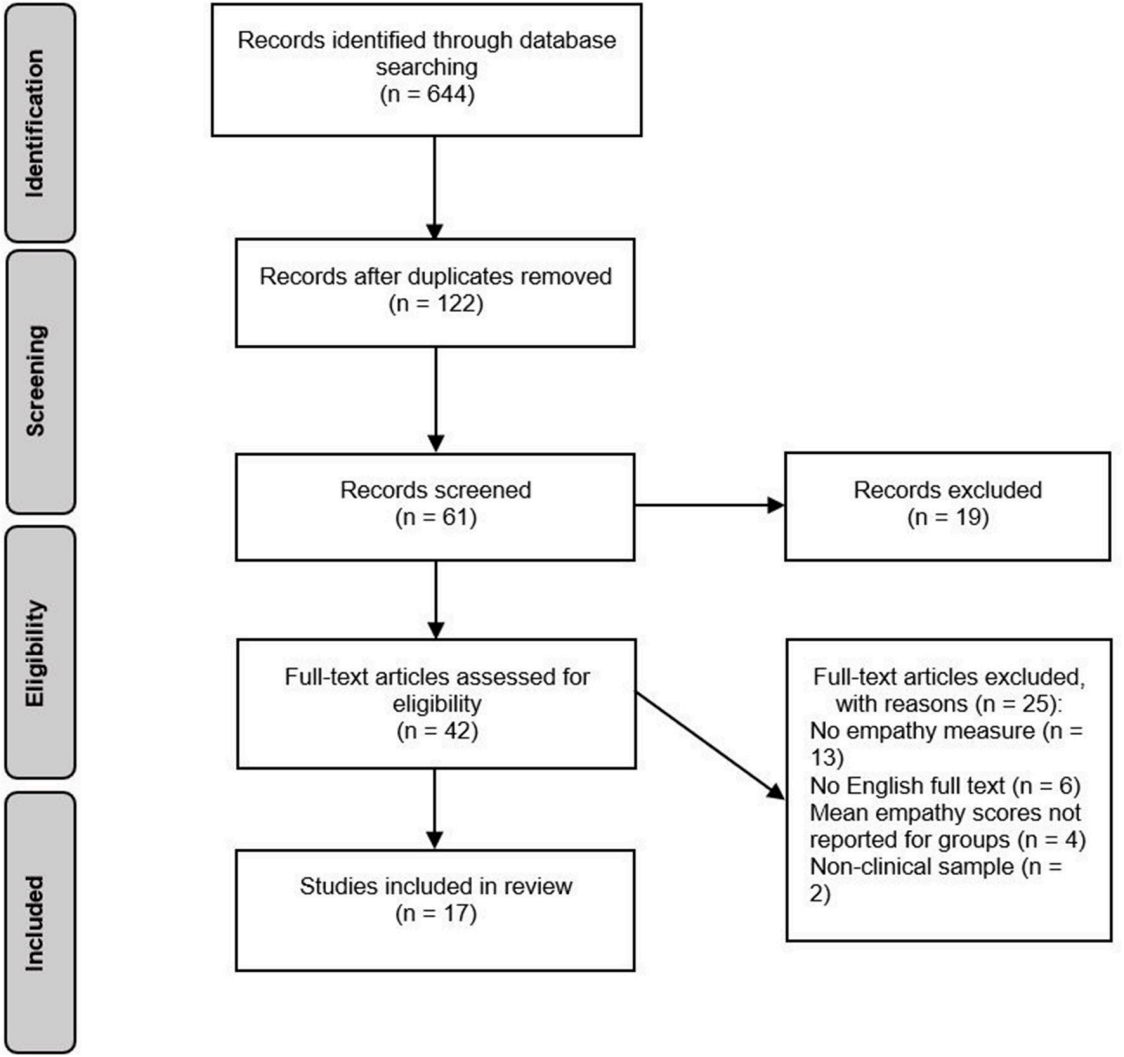
Systematic review search process.

The selection process for studies is displayed in Figure 1. In total, the search generated 644 records. After removing duplicates, 122 records were assessed for relevance based on article titles. If titles were ambiguous or potentially relevant, records were retained and their abstracts screened against the eligibility criteria. This resulted in 61 abstracts being screened, 19 of which were excluded as they did not meet eligibility criteria. After screening of abstracts, 42 potentially eligible full-text articles were identified. One study included a sample of participants with BN, however at the time of publication, BN was not yet included in the DSM. The study was included in the review as participants had a clinical diagnosis of BN. If means and standard deviations for individual groups were not reported, study authors were contacted. If no response was received, studies were excluded. Evaluation of these full texts resulted in 25 studies being excluded, and 17 studies being included in the review.

### Data Extraction

The following data was extracted from each paper that met all eligibility criteria: number of participants in each group, mean age, mean body mass index (BMI), percentage of female participants, empathy measure used, mean empathy scores, and any subscale scores, if they were reported. Where studies reported sub-scale scores only, total, cognitive, and affective empathy scores were calculated so that studies could be included in meta-analyses.

### Data Analysis

All analyses were performed using R Studio (39) using the metafor package (40). Cohen's d was used to estimate effect sizes and is reported with 95% confidence intervals (CIs). Effect sizes are interpreted using Cohen's (41) definitions of small (0.2), medium (0.5), and large (0.8). Negative effect sizes indicate lower empathy scores in the ED group compared to HC. Separate meta-analyses were performed for different components of empathy. Where two measures of empathy were used in the same study (and therefore on the same group of participants), a multivariate meta-analysis was performed using the rma.mv command. Between-study heterogeneity was calculated using Cochran's Q test. Where heterogeneity was found (*p* < 0.05), meta-regressions were performed using age and empathy measure as moderators.

### Risk of Bias

Publication bias was assessed through visual inspection of funnel plots, where the absence of studies in the bottom right corner indicates publication bias. The symmetry of the funnel plots was formally assessed using Begg's rank correlation test (42). Publication bias was also assessed using Rosenthal's fail-safe N (43), which estimates the number of unpublished studies required to change the significant effect size into a non-significant one.

Risk of bias in individual studies was assessed using the Clinical Appraisal Skills Programme checklist for case–control studies (44). The checklist considers how methodological features of studies may have impacted the results, e.g., exclusion and inclusion criteria, recruitment sources, and whether potential confounding variables were included in analyses. Studies can receive a maximum score of 17.

## Results

### Study Characteristics

Study characteristics are shown in Table 1. Fourteen of the included studies compared AN and HC groups. Of these studies, one study also included a recovered AN group, two included an ASD group, and one included a group with BPD. Two studies compared those with binge eating disorder (BED) to HC, and one study compared participants with BN to HC.

**Table 1:** Characteristics of studies.

**References**	**Group**	**Mean age (SD)**	**Mean BMI (SD)**	**% female**	**Empathy measure**	**Mean (SD) total empathy**	**Subscales reported?**	
							**Mean (*SD*) cognitive empathy**	**Mean (*SD*) affective empathy**
Adenzato et al. (45)	30 AN	19.73 (6.06)	15.06 (1.74)	100	EQ	**44.17 (11.47)**	NR	NR
	32 HC	20.47 (2.72)	20.21 (1.45)	100		**50.72 (8.35)**		
Aloi et al. (46)	22 BED	43.8 (10.7)	36.9 (4.2)	81.4	EQ	41.8 (14.9)	NR	NR
	16 sub-threshold BED	42.5 (11.3)	37.5 (4.5)	68.8		50.5 (11.6)		
	20 obese controls	50.6 (8.6)	38.2 (6.5)	45		50.1 (12.4)		
Baron Cohen et al. (47)	66 AN	17.85 (0.39)	NR	100	EQ (adult and adolescent versions)^†^	**Younger: 44.7 (16.4)** Older: 49.6 (9.7)	NR	NR
	1609 HC	18.56 (3.99)	NR	100		**Younger: 51.2 (14.3)** Older: 48.0 (11.3)		
Butler and Montgomery (48)	15 AN	27.9 (9.9)	NR	100	I_7_	15.40 (2.61)	NA	NA
	16 HC	28.4 (8.3)	22.75	100		14.19 (2.74)		
Calderoni et al. (49)	32 AN	14.78 (1.75)	15.07 (1.54)	100	IRI	*5.13 (6.98)*	**0.44 (6.87)**	4.69 (7.08)
	41 HC	14.02 (1.69)	NR	100		*9.44 (5.66)*	**5.24 (6.45)**	4.20 (4.75)
Courty et al. (50)	15 AN	23.9 (4.7)	16.4 (1.7)	93.33	EQ-short	23.0 (6.8)	NR	NR
					IRI	*70.8 (4.83)*	*34.1 (4.85)*	*36.7 (4.8)*
	15 HC	24.0 (4.9)	21.0 (1.8)	93.33	EQ-short	21.1 (7.4)	NR	NR
					IRI	*73.09 (3.79)*	*38.3 (3.31)*	*35.6 (4.22)*
	15 ASD	28.1 (7.5)	23.2 (5.0)	13.33	EQ-short	10.1 (5.7)	NR	NR
					IRI	*65.5 (4.77)*	*32.6 (4.73)*	*32.9 (4.81)*
	15 HC	28.1 (7.3)	22.2 (3.0)	13.33	EQ-short	19.9(3.4)	NR	NR
					IRI	*67.0 (3.39)*	*34.7 (2.75)*	*32.3 (3.93)*
Duchesne et al. (51)	60 BED	NR	38.1	100	IRI	*81.4 (4.04)*	*42.4 (4.40)*	*39.0 (3.65)*
	60 obese controls	NR	37.9	100		*81.3 (3.95)*	*42.7 (3.36)*	*38.6 (4.46)*
	54 HC	NR	21.4 (1.6)	100		*80.6 (4.02)*	*43.4 (4.30)*	*37.2 (3.72)*
Feldman and Eysenck (52)	45 BN	25.13 (6.59)	NR	100	I_7_	14.73 (3.17)	NA	NA
	761 HC	NR	NR	100		14.39 (2.87)		
Gramaglia et al. (53)	39 AN	30.59 (3.0)	16.3	NR	IRI	*82.93 (3.81)*	*41.19 (4.48)*	*41.74 (2.99)*
	48 HC	33.19 (3.37)	21.82	100		*80.48 (3.78)*	*41.9 (4.15)*	*38.58 (3.37)*
Guttman and Laporte (54)	28 AN	22	NR	100	IRI	*72.7 (5.60)*	*35.1 (5.51)*	*37.6 (5.69)*
	26 BPD	32	NR	100		*78.9 (5.45)*	*34.7 (5.56)*	*44.2 (5.35)*
	27 HC	21	NR	100		*71.9 (4.83)*	*35.9 (4.61)*	*36 (5.04)*
Hambrook et al. (55)	22 AN	26.73 (4.77)	15.27 (1.22)	100	EQ	45.9 (12.5)	NR	NR
	45 HC	32.51 (9.63)	23.36 (3.76)	100		46.2 (11.1)		
Jermakow and Brzezicka (56)	11 AN	26.80 (4.3)	NR	100	EQ	44.60 (8.58)	NR	NR
					IRI	**63.10 (3.39)**	*34.9 (6.22)*	*28.2 (4.46)*
	33 female HC	21.33 (1.4)	NR	100	EQ	42.42 (9.84)	NR	NR
					IRI	**70.03 (2.13)**	*38.52 (4.40)*	*31.52 (4.40)*
	10 ASD	28.30 (9.5)	NR	0	EQ	30.00 (5.05)	NR	NR
					IRI	57.90 (2.20)	*33.5 (5.59)*	*24.4 (3.64)*
	27 male HC	21.76 (2.0)	NR	0	EQ	32.63 (9.97)	NR	NR
					IRI	62.70 (2.33)	*33.38 (5.60)*	*29.33 (5.21)*
Lule et al. (57)	15 AN	16.2 (1.26)	17.07 (1.44)	100	IRI	121.14 (11.25)	NR	NR
	15 HC	16.5 (1.09)	21.06 (1.57)	100		118.50 (10.20)		
Morris et al. (58)	28 AN	26.3 (7.9)	15.5 (1.3)	100	SEQ	**18.8 (2.5)**	NA	NA
	25 AN-REC	29.5 (9.2)	20.1 (1.9)	100		19.8 (3.0)		
	54 HC	29.4 (9.6)	23.1 (3.9)	100		**20.4 (2.4)**		
Nandrino et al. (59)	23 AN	19.64 (1.82)	15.2 (1.07)	100	BES	79.57 (6.70)	35.57 (3.45)	44.00 (5.44)
	23 HC	20.65 (1.90)	21.05 (1.78)	100		80.78 (6.04)	36.78 (3.19)	44.00 (4.93)
Peres et al. (60)	41 AN	16.2 (1.4)	79.78 (8.71) %IBW	100	IRI	*74.44 (4.30)*	35.5 (6.99)	**39.0 (6.45)**
	38 HC	15.84 (1.83)	100.5 (11.71) %IBW	100		*73.1 (4.1)*	37.6 (7.18)	**35.6 (5.21)**
Redondo and Herrero-Fernandez(61)	38 AN	21.9 (5.30)	NR	100	EQ-short	23.42 (7.25)	11.26 (4.84)	7.11 (2.68)
					IRI^††^	NR	NR	NR
	321 HC	NR	NR	100	EQ-short	25.79 (7.21)	11.03 (4.63)	7.55 (2.35)
					IRI^††^	NR	NR	NR

*Significant differences between ED and HCs are indicated in bold. Italics indicate where scores were not reported in the study, but could be calculated from subscale scores. Potential significant differences could therefore not be reported for calculated scores. AN, anorexia nervosa; AN-REC, recovered anorexia nervosa; ASD, autism spectrum disorder; BED, binge eating disorder; BES, Basic Empathy Scale; BMI, body mass index; BN, bulimia nervosa; BPD, borderline personality disorder; EQ, Empathy Quotient; HC, healthy control; I_7_, Impulsiveness, Venturesomeness, and Empathy questionnaire; IRI, interpersonal reactivity index; NA, not applicable; NR, not reported; SD, standard deviation; SEQ, Socio-Emotional Questionnaire*.

† *Groups were split into groups depending on age and EQ version used*.

†† *Only the PT subscale of the IRI was used*.

In total, 6 different self-report measures were used across studies, with the Interpersonal Reactivity Index [IRI; (62)] being used most often (9 studies). The IRI comprises of four subscales: perspective taking (PT; the tendency to spontaneously adopt the psychological viewpoint of others), fantasy (FS; the tendency to identify oneself with fictional characters in books, plays and movies), empathic concern (EC; assesses “other-oriented” feelings of sympathy and concern for others), and personal distress (PD; assesses “self-oriented” feelings of anxiety and unease in tense interpersonal settings). Cognitive and affective empathy scores can be calculated by taking the sum of PT and FS, and EC and PD respectively. The Empathy Quotient [EQ; (6)], and the EQ-short (63) were used in seven studies, and both have three subscales: cognitive empathy, affective empathy, and social skills. Other measures used were: the empathy subscale of the Impulsiveness, Venturesomeness, and Empathy questionnaire (I_7_; (64) (2 studies), the empathy subscale of the Socio-Emotional Questionnaire [SEQ; (65)] (1 study), and the Basic Empathy Scale [BES; (66)] (1 study). One study used two different versions of the EQ depending on participants' age; the parent reported version for younger adolescents, and the self-report version for older adolescents (47). Only the self-report scores are included in the meta-analysis, as this was the focus of the present review.

Methodological quality of the studies varied considerably (range: 7–16). None of the studies reported a power calculation, and sample sizes were generally small (ranging from 11 to 66 in ED groups). All but one study (46) matched participants on at least one characteristic, most often sex. The mean age of participants ranged from 14.02 to 50.60 years, although three studies did not report the mean age of at least one participant group (51, 52, 61). Seven studies did not report mean BMI or percentage IBW in at least one participant group (47–49, 52, 54, 56, 61). Most studies used exclusively female samples, however three studies included male participants (46, 50, 56).

### Synthesized Findings

**Figure 2:**
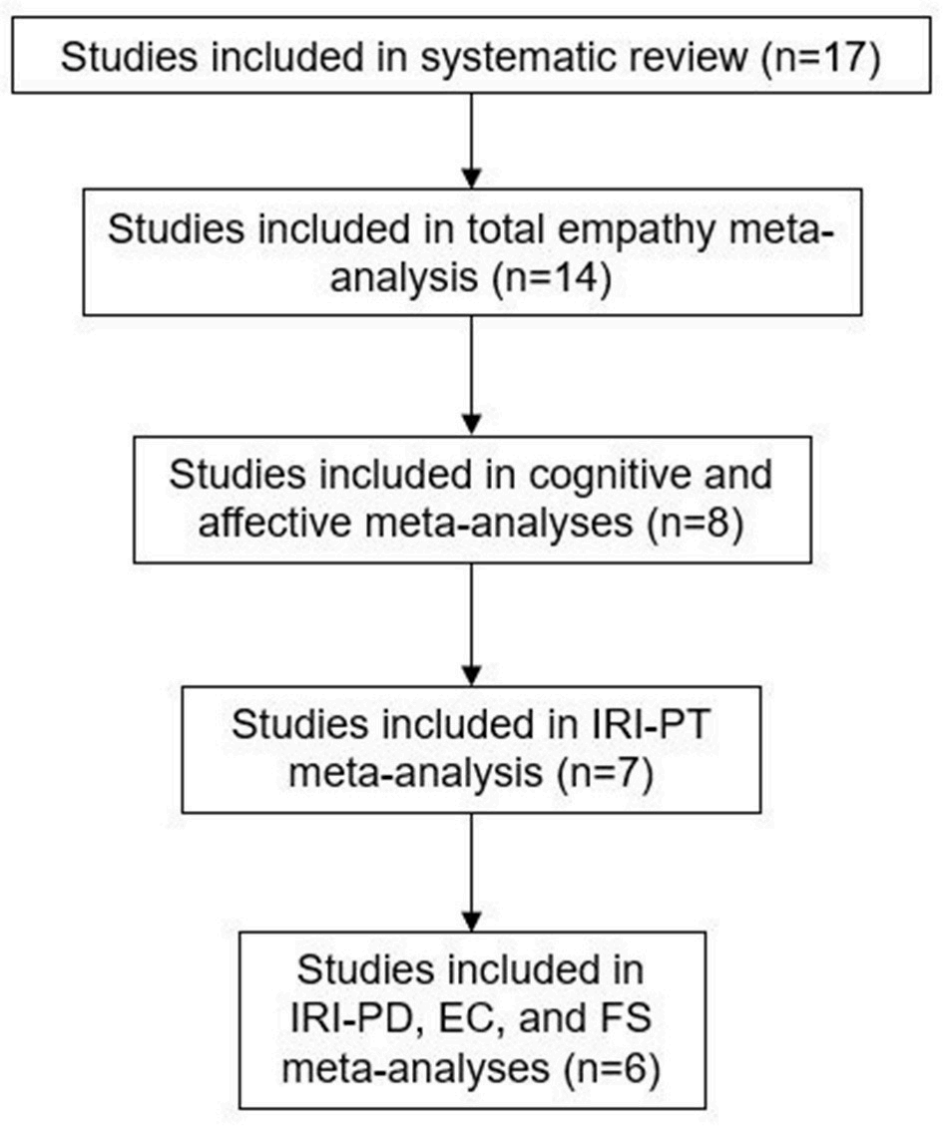
Studies included in the review and meta-analyses.

Only studies comparing AN and HC could be included in meta-analyses, due to too few studies with other ED groups (2 BED, 1 BN). The number of studies in each meta-analysis is displayed in Figure 2.

#### Total Empathy

**Figure 3:**
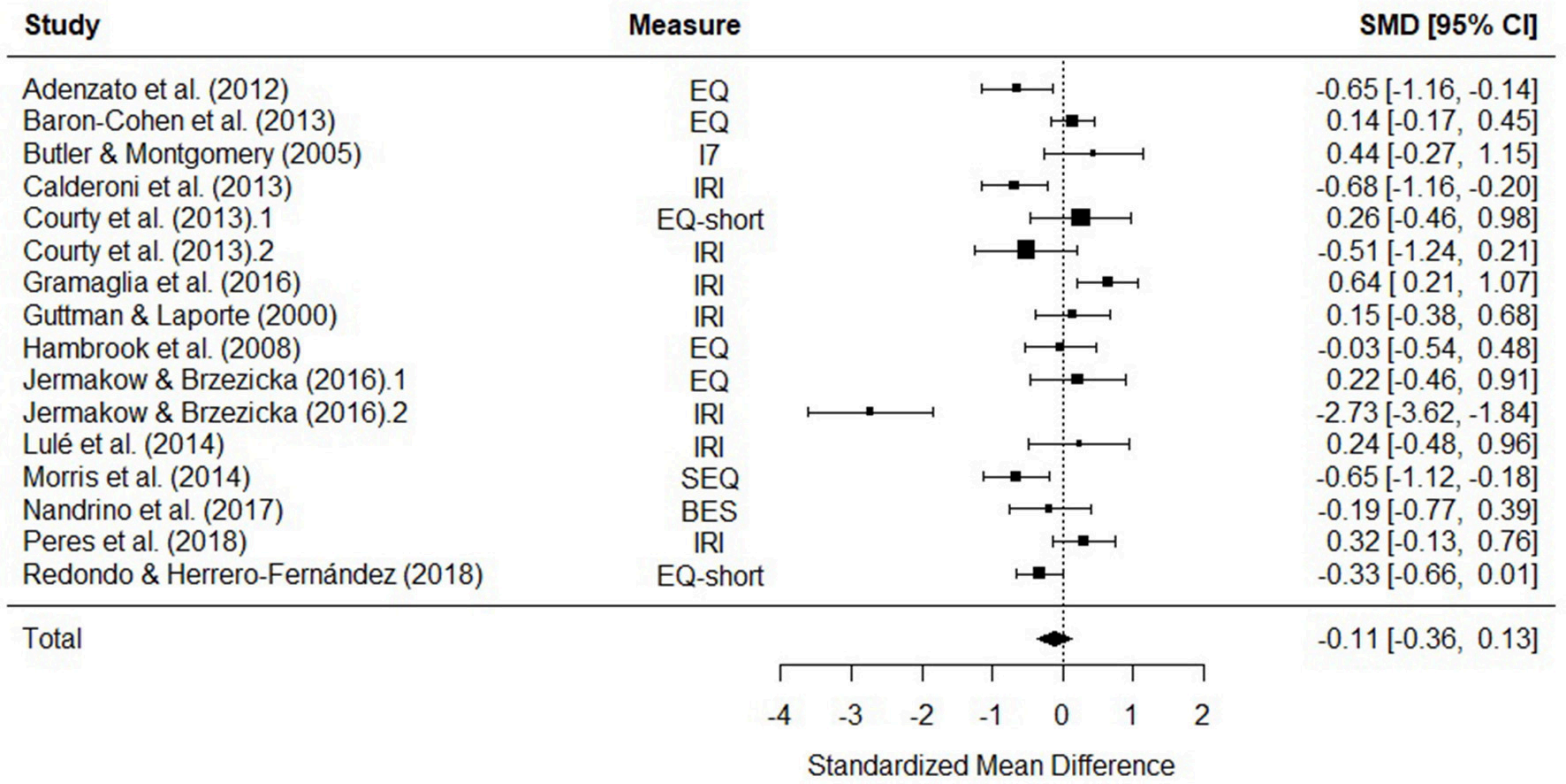
Forest plot of standardized mean effect size for differences (SMD) between anorexia nervosa (AN) and healthy controls (HC) on total empathy scores. Negative effect sizes indicate lower empathy scores in the AN group. BES, Basic Empathy Scale; CI, confidence interval; EQ, empathy quotient; I_7_, Impulsiveness, Venturesomeness, and Empathy questionnaire; IRI, interpersonal reactivity index; SEQ, Socio-Emotional Questionnaire.

Fourteen studies were included in a meta-analysis comparing total empathy scores in AN and HCs. The random effects model with a total sample size of 2165 participants (AN = 379, HC = 1746) revealed that total empathy scores in AN did not differ from those of HCs [*d* = −0.11, (95% CI −0.36, 0.13) *z* = −0.92, *p* = 0.36] (Figure 3).

There was evidence of significant heterogeneity across studies [*Q*_(15)_ = 79.61, *p* < 0.001], therefore meta-regressions with age and empathy measure as moderator variables were performed. The moderators explained a significant amount of the variance [*QM*_(6)_ = 27.88, *p* = < 0.001], however no single factor had a significant influence on the size of the effect. The test for residual heterogeneity was significant [*QE*_(8)_ = 65.08, *p* = < 0.001].

#### Cognitive Empathy

**Figure 4:**
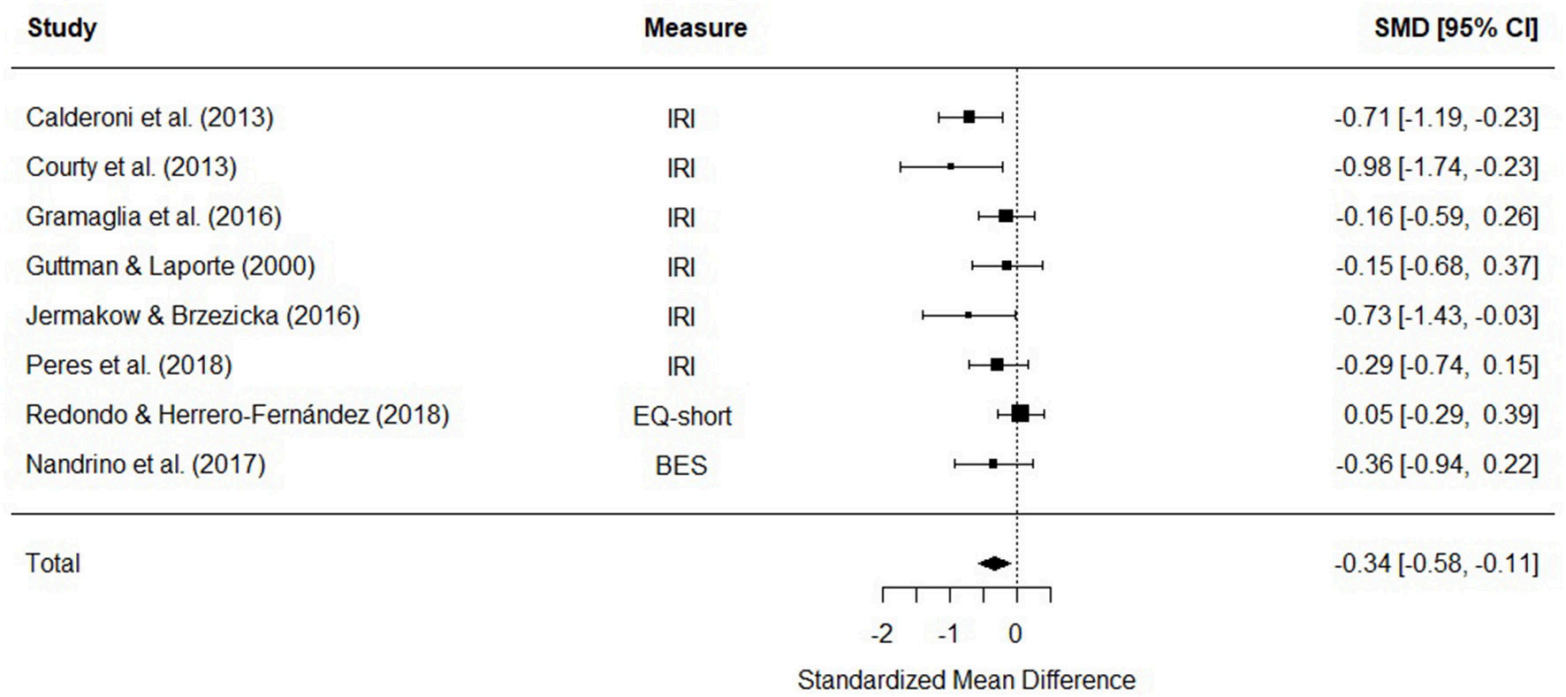
Forest plot of standardized mean effect size for differences (SMD) between anorexia nervosa (AN) and healthy controls (HC) on cognitive empathy scores. Negative effect sizes indicate lower empathy scores in the AN group. BES, Basic Empathy Scale; CI, confidence interval; EQ, empathy quotient; IRI, interpersonal reactivity index.

Eight studies were included in a meta-analysis comparing cognitive empathy scores in AN and HC. The random effects model with at total sample size of 773 participants (AN = 227, HC = 546) revealed that cognitive empathy scores in AN were significantly lower than HCs [*d* = −0.34, (95% CI −0.58, −0.11) *z* = −2.86, *p* = 0.004] (Figure 4). There was no evidence of significant heterogeneity [*Q*_(7)_ = 12.27, *p* = 0.09].

#### Affective Empathy

**Figure 5:**
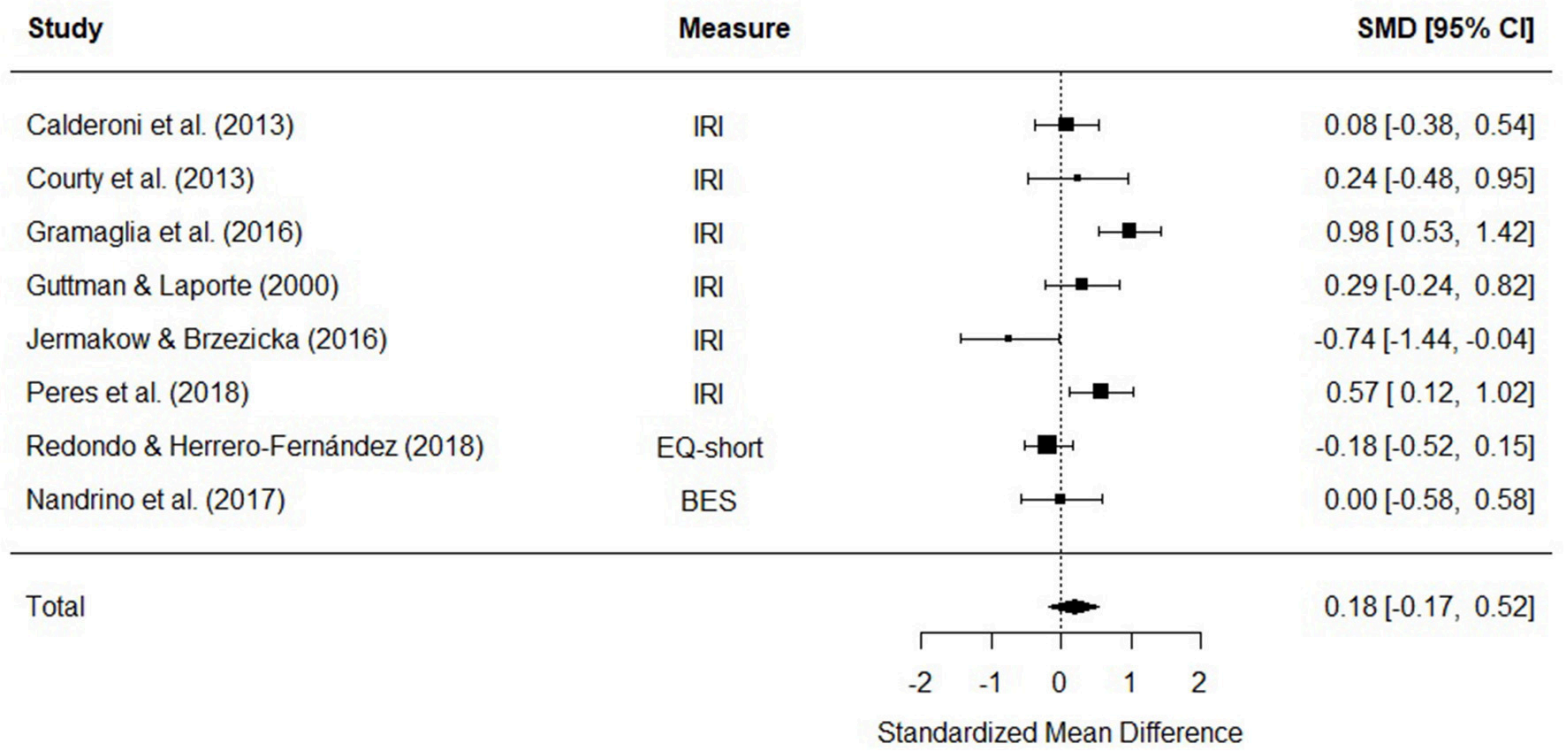
Forest plot of standardized mean effect size for differences (SMD) between anorexia nervosa (AN) and healthy controls (HC) on affective empathy scores. Negative effect sizes indicate lower empathy scores in the AN group. BES, Basic Empathy Scale; CI, confidence interval; EQ, empathy quotient; IRI, interpersonal reactivity index.

Eight studies were included in a meta-analysis comparing affective empathy scores in AN and HC. The random effects model with a total sample size of 773 participants (AN = 227, HC = 546) revealed that affective empathy scores in AN did not differ from those of HCs [*d* = 0.18, (95% CI −0.17, 0.52) *z* = 1.01, *p* = 0.31] (Figure 5).

There was evidence of significant heterogeneity across studies [*Q*_(7)_ = 26.99, *p* < 0.001], therefore meta-regressions with age and empathy measure as moderator variables were performed. The moderators did not explain a significant amount of the variance [*QM*_(3)_ = 0.64, *p* = 0.88], and the test for residual heterogeneity was significant [*Q*_(4)_ = 17.6, *p* = 0.002].

### Risk of Bias

**Figure 6:**
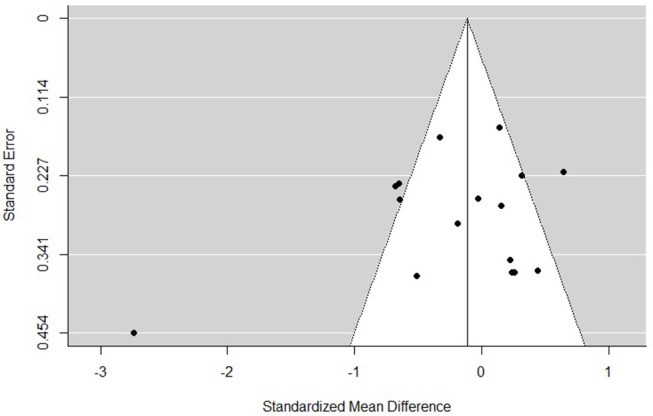
Funnel plot of studies included in the total empathy meta-analysis.

**Figure 7:**
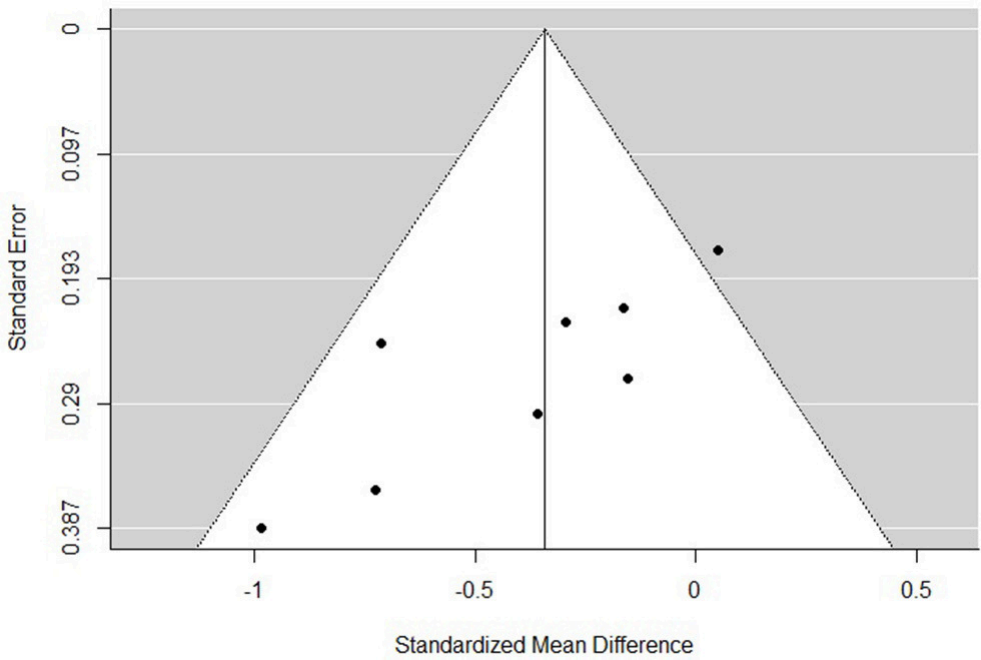
Funnel plot of studies included in the cognitive empathy meta-analysis.

**Figure 8:**
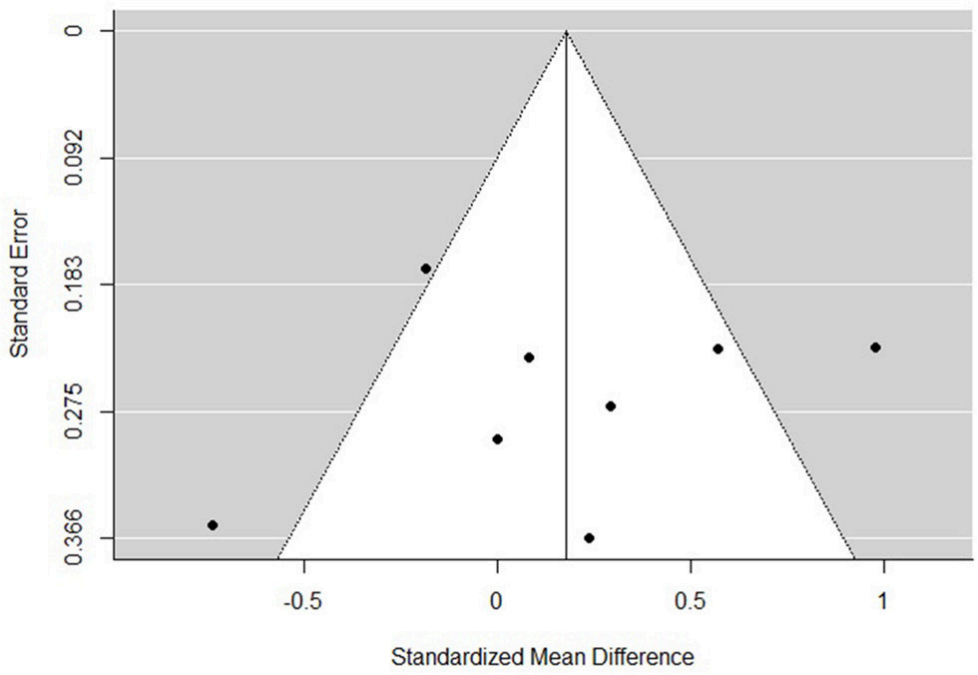
Funnel plot of studies included in the total affective meta-analysis.

The funnel plots for total empathy, cognitive empathy, and affective empathy scores are displayed in Figures 6–8. There was no evidence of publication bias in the total empathy meta-analysis (Begg's test *p* = 0.45), however there was evidence of publication bias in the studies included in the cognitive empathy meta-analysis (Begg's test *p* = 0.03, Rosenthal's fail safe *N* = 38). Studies included in the affective empathy meta-analysis did not show any evidence of publication bias (Begg's test *p* = 0.40).

### Additional Analyses

Because several studies reported on the PT, FS, EC, and PD subscales of the IRI, additional meta-analyses were performed to test for differences between AN and HC. Six studies reported scores for all four subscales, while one additional study reported PT scores only. The results are shown in Table 2. AN had significantly lower FS scores compared to HC, however there were no significant differences in the other sub-scales. There was no evidence of significant heterogeneity in any of the subscale meta-analyses, nor was there significant evidence of publication bias (Begg's test all *p* > 0.05) (see Supplementary Material for subscale forest and funnel plots).

**Table 2:** Statistical outcomes for meta-analyses of the four IRI subscales.

**IRI subscale**	**N studies**	**Pooled AN sample N**	**Pooled HC sample N**	**Cohen's *d***	**95% CI**	***Z***	***p***
Perspective taking	7	204	523	−0.2	−0.44, 0.05	−1.59	0.11
Fantasy	6	166	202	**−0.41**	−0.62, −0.20	3.83	**>0.001**
Empathic concern	6	166	202	0.01	−0.20, 0.22	1.1	0.92
Personal distress	6	166	202	0.3	−0.13, 0.74	1.36	0.17

*Significant differences between AN and HCs are indicated in bold. AN, anorexia nervosa; CI, confidence intervals; HC, healthy control; IRI, Interpersonal Reactivity Index*.

### Qualitative Findings

#### Studies in AN

Studies using the EQ or the EQ-short reported very mixed findings. Adenzato et al. (45) found that those with AN had significantly lower total EQ scores compared to HCs. In adolescents, this was only found to be true for those aged 12–15years, using the parent report version of the EQ (47). The older AN group did not differ from age-matched HC on the self-report EQ. Redondo and Herrero-Fernández (61) found that while total EQ-short scores in those with AN and HCs did not differ, those with AN scored significantly lower than HCs on the social skills subscale. Three studies found no differences in EQ scores between AN and HC, however both groups scored significantly higher than those with ASD (50, 55, 56).

Results from studies using the IRI were similarly mixed. Only two studies tested for group differences in total IRI scores, with one reporting significantly lower scores in those with AN than HCs (56) and the other reporting no differences (57). Two studies tested for group differences in cognitive and affective empathy sub-scores of the IRI. Cognitive empathy scores are calculated by summing the F and PT subscale scores together, while the EC and PD subscale scores are summed to calculate affective empathy scores. Calderoni et al. (49) found that those with AN had significantly lower cognitive empathy scores, whereas Peres et al. (60) reported significantly higher emotional empathy scores in AN compared to HC.

Six studies reported on group differences between AN and HCs on IRI EC, PD, FS, and PT (with one additional study included the PT subscale only). Regarding EC, there were no significant differences between AN and HC across all six studies (49, 50, 53, 54, 56, 60). However, those with AN had significantly higher EC scores compared to those with ASD (50), and significantly lower scores than women with BPD (54). Two studies found that those with AN scored higher on PD than HC (53, 60), while one reported that AN and ASD groups had lower scores than HCs (56). Three studies reported no differences in PD scores between AN and HC, however those with BPD had higher scores than both AN and HC groups (49, 50, 54). Regarding the FS subscale, it was found that those with AN had significantly lower scores than HC, similar to those with ASD (49, 50). However, four studies did not find significant differences between groups (53, 54, 56, 60). Calderoni et al. (49) and Redondo and Herrero-Fernandez (61) reported that AN had significantly lower PT scores compared to HCs, however the remaining five studies found no significant differences (50, 53, 54, 56, 60).

The remaining AN studies used the I_7_, the empathy subscale of the SEQ, and the BES. Morris et al. (58) found that AN scored significantly lower on the SEQ than HC. Scores in the recovered AN group did not differ from either group, lying between the two. The remaining two studies found no significant differences between AN and HCs (48, 59). However, both studies were limited in their sample sizes (15 and 23 participants in the clinical groups respectively), and therefore there may not be sufficient power to detect group differences.

#### Studies in Other EDs

Only three studies involved participants with BED or BN. Feldman and Eysenck (52) reported no differences in empathy scores between women with BN and HCs. However, this study had the poorest methodological quality rating of all studies include in the review, mainly because it included little information about the HC group, and did not control for any confounding variables. In BED, total empathy scores did not significantly differ across those with BED, subthreshold BED, and HCs (46). However, 51 reported that women with BED scored significantly higher than obese and HC women on the PD subscale of the IRI. Further, a logistic regression revealed that lower PT and higher PD scores were associated with BED. Unfortunately, this study did not control for confounding variables such as depression, which has been found to be associated with PD (9).

#### Associations With Psychopathology and Clinical Variables

Few studies examined associations between empathy and clinical variables or other measures of psychopathology. In BED and AN, negative correlations were found between EQ and alexithymia scores on the twenty-item Toronto Alexithymia Scale [TAS-20; (67)], such that lower levels of empathy were associated with higher alexithymia (45, 46). The latter study also found that higher EQ scores were associated with more social support in AN, as measured by the Multidimensional Scale of Perceived Social Support [MSPSS; (68)]. Only two studies examined whether empathy was associated with ED psychopathology and illness severity in AN. Baron-Cohen et al. (47) reported that EQ scores were not associated with scores on the Eating Disorder Examination Questionnaire [EDEQ; (69)], and Calderoni et al. (49) found that cognitive empathy scores were not associated with BMI, disease duration, or general psychopathology in AN. Finally, Peres et al. (60) reported that IRI, AE and PD subscale scores were positively associated with anxiety, but not depression, as measured by the Hospital Anxiety and Depression Scale [HADS; (70)]. However, linear regressions revealed that anxiety did not explain the differences in empathy between AN and HC better than group membership.

## Discussion

### Summary of Main Findings

The aim of this review was to examine group differences in empathy in those with EDs compared to HC, and provide a qualitative synthesis of the literature. Meta-analyses were run for total empathy, cognitive empathy, affective empathy, and four further sub-components of empathy: PT, FS, EC, and PD. There were no significant differences between those with AN and HC in overall empathy (14 studies) or affective empathy scores (8 studies). However, it was found that those with AN had significantly lower cognitive empathy scores compared to HC (8 studies), with a small effect size. Further, it those with AN had significantly lower FS scores than HC (6 studies), with a small effect size, but did not significantly differ from HC on any of the other IRI subscores.

The finding that AN have lower cognitive empathy abilities compared to HC is in accordance with studies examining related, performance-based measures of empathy, such as ToM (32), emotion recognition (31), and emotional intelligence (71). Affective empathy has been less well-studied in EDs, although it appears from this review, and a few experimental studies, that individuals with ED are not impaired in affective empathy. For example, one study found that those with BN reported higher levels of sadness than restrained eaters and HCs in response to video clip, during which they were asked to identify themselves with the protagonist whose boyfriend leaves them for an attractive woman (72). Another study examined individuals' own emotional reactions to video clips depicting an individual displaying emotion, finding that the intensity of the emotions experienced by those with EDs (AN and BN) did not differ from HC (73). However, those with EDs did show less facial expressivity while watching the clips—a component of empathy that has been termed “motor empathy” (74). Studies that utilize physiological measurements of empathy, such as facial electromyographic activity (EMG), skin conductance, and heart rate may be useful in further understanding affective empathy in EDs.

There are a number of possible explanations for the dissociation between cognitive and affective empathic abilities found here. Distinct brain systems for cognitive and affective empathy have been described: the ventromedial prefrontal cortex is involved in cognitive empathy, while the inferior frontal gyrus is involved in affective empathy (75). Neuroimaging studies have reported differences in the ventromedial prefrontal cortex in those with AN (76, 77), thus providing a possible explanation for lowered cognitive empathy abilities. fMRI studies utilizing performance-based measures of empathy could be useful in testing this hypothesis. Relatedly, difficulties in executive functioning are reported in those with AN and BN (78). Since executive functions contribute to the development of cognitive empathy (79), it would be of interest to determine whether there is a relation between empathy abilities and executive functioning in those with EDs. Relatedly, it might be that reduced attention to faces and eyes found in AN (28, 80, 81) leads to decreased cognitive empathy abilities.

There was evidence of significant heterogeneity in the overall empathy and affective empathy studies. While age and empathy measurement did explain some of the variance in total empathy scores, no single factor had a significant influence on the size of the effect. Due to a lack of studies reporting on factors such as BMI and illness duration, it was not possible to include these indicators of illness severity as moderators. The two studies that did examine potential associations between ED severity and empathy did not find any significant relationships (47, 49). Research examining the relationship between illness severity and constructs related to empathy such as mentalizing (the ability to understand the mental states of oneself or others, and how such states might influence behavior) have been mixed. While some have reported independence from BMI and illness length (82), a meta-analysis found that poorer performance on the RMET was associated with longer illness duration (83). Examining whether cognitive or affective empathy are state or trait variables will be important in characterizing the socio-emotional phenotype proposed for EDs (84).

Relatedly, it would be of interest to examine whether other psychopathological variables may have influenced the effect sizes reported in this review. One candidate is ASD symptoms. Support for this idea comes from a longitudinal population-based study which examined mentalizing abilities in those with AN and HCs (21), in which 29% of the AN group also met criteria for a diagnosis of ASD. They found that when mentalizing ability was compared between AN+ASD, AN only, and HCs, only the AN+ASD group had significantly lower scores than HC. Thus, it is possible that there is a sub-group of individuals with AN who display the most severe difficulties in socio-emotional measures, whose difficulties are missed when assessing group differences. While ASD symptoms could not be included as moderators in the meta-analyses presented here, it would be important to ascertain whether reduced empathy in AN is a characteristic of the ED, or some other comorbid psychopathology.

Alternatively, it could be the case that the heterogenous results in AN might be explained by alexithymia. Indeed, a few studies included in this review found that lower levels of empathy in AN and BED were associated with higher alexithymia (45, 46). Alexithymia is a subclinical phenomenon characterized by difficulties in describing and recognizing one's own emotions, and distinguishing feelings from bodily sensations of emotional arousal. “Shared network” models of empathy propose that the networks in the brain responsible for processing one's own emotions are the same networks used to represent the emotions of others (85–87). Thus, it is possible that the high levels of alexithymia experienced by those with AN might be responsible for lower levels of empathy compared to HCs. In support of this hypothesis, an fMRI study in ASD showed that the strength of empathic brain responses in the left anterior insula were predictive of degree of alexithymia in both ASD and HCs, but did not vary as a function of group (88). The potential contribution of alexithymia to reduced empathy, and indeed other aspects of socio-emotional functioning in EDs, should be explored.

Only two studies examined empathy in BED, finding no difference in total empathy scores, but significantly higher PD scores compared to HCs (46, 51). The finding that those with BED experience more stress and unease in tense social settings is consistent with literature documenting emotion regulation difficulties in those with BED, and it is hypothesized that binge eating may be a strategy to deal with increased negative emotions (89). It would therefore be of interest to examine whether higher PD scores in BED are associated with more severe ED psychopathology. The only study that measured empathy in BN found no significant differences in empathy compared to HCs (52). This study used the I_7_ to measure empathy, and therefore no study has yet examined cognitive and affective components of empathy in BN. Clearly, the lack of studies in BN and BED prevent any conclusions being made regarding empathy in these groups. Given that problems with interpersonal functioning are a prominent feature in BN (18, 90), research using multidimensional measures of empathy in this population are needed.

The findings from the current review have implications for treatment of AN. Socio-communicative and interpersonal problems are associated with poorer outcomes (20, 21, 82, 91, 92) and more severe ED psychopathology (36, 37), therefore socio-emotional functioning may be a potential target for the development of new, more holistic treatment approaches. For example, group social skills interventions are effective in improving communication, social anxiety, and social functioning in those with ASD (93, 94). There is also evidence to suggest that Cognitive Remediation and Emotion Skills Training (CREST), an intervention designed to improve emotion processing, is effective in decreasing alexithymia and social anhedonia, while increasing motivation in those with AN (95, 96). Recently, there has also been interest in exploring the effect of oxytocin, a hormone implicated in prosocial behavior, on socio-emotional functioning (97, 98). In ASD, administration of intranasal oxytocin has been found to increase interactions with socially cooperative peers, and enhance feelings of trust (99). Oxytocin also increased participants' attention to the eyes of pictures of faces, avoidance of which is a core feature of ASD (100). A few studies have examined the effect of oxytocin on socio-emotional cognition in those with EDs. One study found intranasal oxytocin increased emotion recognition and decreased calorie consumption in those with BN, however no effects were seen in AN (101). Another found no effect of oxytocin on RMET performance in AN (102). However, whether oxytocin has an effect on real-life social behavior in those with EDs has yet to be examined.

### Limitations

Several limitations of this review should be noted. Firstly, many studies did not report empathy subscale scores, and therefore could not be included in affective and cognitive empathy meta-analyses. Secondly, although this method has been employed in previous reviews of this type (103, 104), it could be questioned whether it is appropriate to compare different scales that purport to measure the same empathy constructs. For example, the affective subscales of the IRI have been criticized as more closely reflecting sympathy, as they focus on reactions to others, rather than emotion matching (105). However, studies in this review generally included the most widely used measures of empathy (e.g., the EQ and the IRI), and as previously noted, empathy measure did not significantly influence effect sizes in moderator analyses.

It is also important to note the limitations of self-report empathy measures generally. Socially desirable responding may be an issue with self-report measures, as they do not objectively measure empathic abilities, but rather how empathetic individuals perceive themselves to be. In other psychiatric disorders, a discrepancy between performance-based empathy tasks and self-report measures has been reported. For example, a meta-analysis found that people with schizophrenia display greater affective empathy deficits in performance-based tasks than on self-report measures (103). If affective empathy partly relies on one's ability to report on their own emotional reactions, this might be especially difficult in populations with high levels of alexithymia, such as AN (106).

The number of studies in other EDs, such as BN and BED, was greatly lacking. Therefore, meta-analyses for group differences between these groups and HCs could not be carried out. Furthermore, only three studies included males with EDs, thus the results from this review cannot be generalized to this population. Interestingly, it is reported that while males with EDs (AN, BN, or eating disorder not otherwise specified) show the same difficulties in cognitive flexibility and weak central coherence often found in women with EDs, they do not differ from HC men in terms of ToM performance or sensitivity to social threat (107). Future work should therefore examine performance in a broader range of socio-emotional tasks in order to understand possible similarities and differences in the male and female presentations of EDs.

Finally, there was evidence of publication bias in the cognitive empathy meta-analysis, indicating that studies with non-significant results may have been missing from analyses. However, the fact that the affective empathy meta-analysis, which included the same studies as the cognitive meta-analysis, did not show any evidence of publication bias and showed a non-significant result, perhaps lends support to the validity of our findings. Nonetheless, the results should be interpreted with caution.

## Conclusions

Although there is an extensive literature documenting difficulties in ToM and emotion recognition in those with EDs, relatively little is known about empathic abilities in this population. This systematic review and meta-analysis aimed to examine whether those with EDs differed from HCs on several dimensions of self-reported empathy, and provide a qualitative synthesis of the literature. While those with AN did not differ from HCs in overall empathy, a meta-analysis of 8 studies found that AN had significantly lower levels of cognitive empathy compared to HC, with a small effect size. It was also found that AN had significantly lower levels of fantasy, a subdivision of cognitive empathy. AN did not differ from HC in affective empathy. This profile of intact affective empathy and lowered cognitive empathy mirrors that of those with ASD, a disorder that shares a number of neuropsychological and socio-cognitive traits with AN. Conclusions regarding the empathic profiles of those with other EDs are not possible, given the lack of studies in these groups. Future research should investigate empathic abilities in other EDs, and examine the influence of comorbid psychopathological traits.

## Author Contributions

JK-G performed the search, data extraction, and wrote the manuscript. KT leads the research group within which this work was conducted and is JK-G lead supervisor for Ph.D. KT and AH edited the manuscript before submission.

### Conflict of Interest Statement

The authors declare that the research was conducted in the absence of any commercial or financial relationships that could be construed as a potential conflict of interest.
