# Supplementary material for: Cognitive and Affective Empathy in Eating Disorders: A Systematic Review and Meta-Analysis
Source: Front Psychiatry. 2019 Mar 4;10:102. doi: 10.3389/fpsyt.2019.00102 (PMC6410675; doi:10.3389/fpsyt.2019.00102)
Supplement: Supplementary file 1 [file Data_Sheet_1.docx]

**Supplementary material**


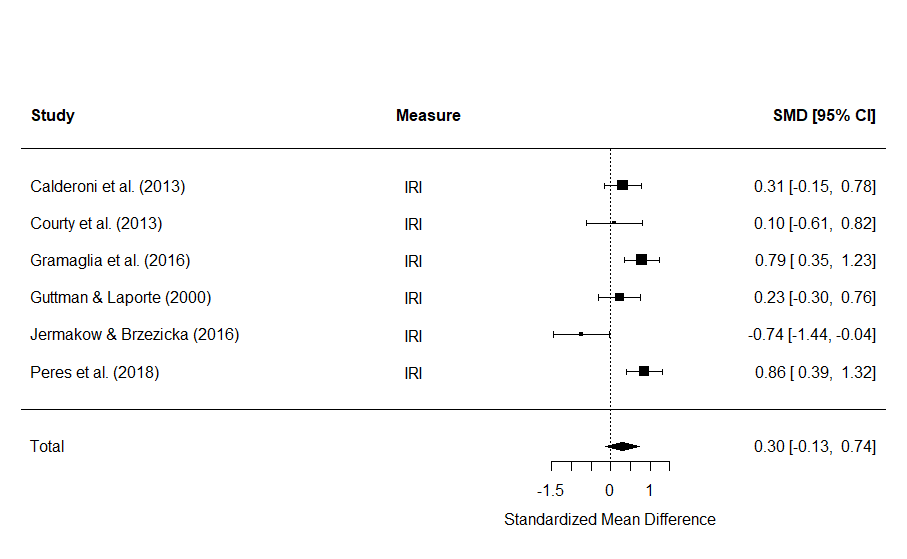


Figure A. Forest plot of standardized mean effect size for differences (SMD) between AN and HC on the personal distress subscale of the IRI. Negative effect sizes indicate lower empathy scores in the AN group.


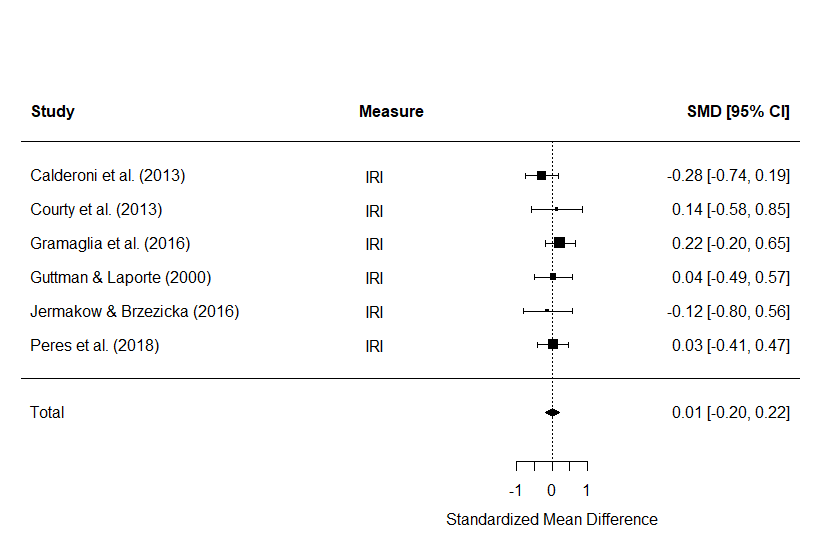


Figure B. Forest plot of standardized mean effect size for differences (SMD) between AN and HC on the empathic concern subscale of the IRI. Negative effect sizes indicate lower empathy scores in the AN group.


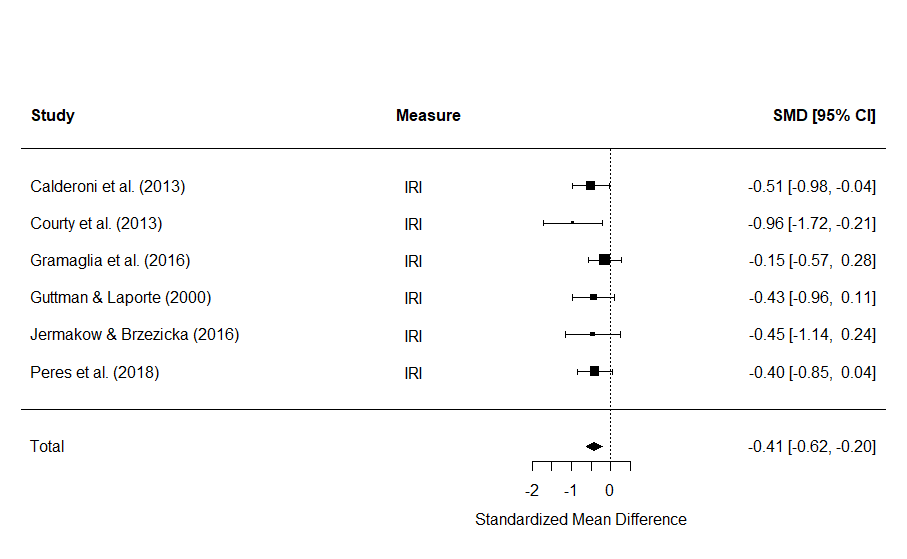


Figure C. Forest plot of standardized mean effect size for differences (SMD) between AN and HC on the fantasy subscale of the IRI. Negative effect sizes indicate lower empathy scores in the AN group.


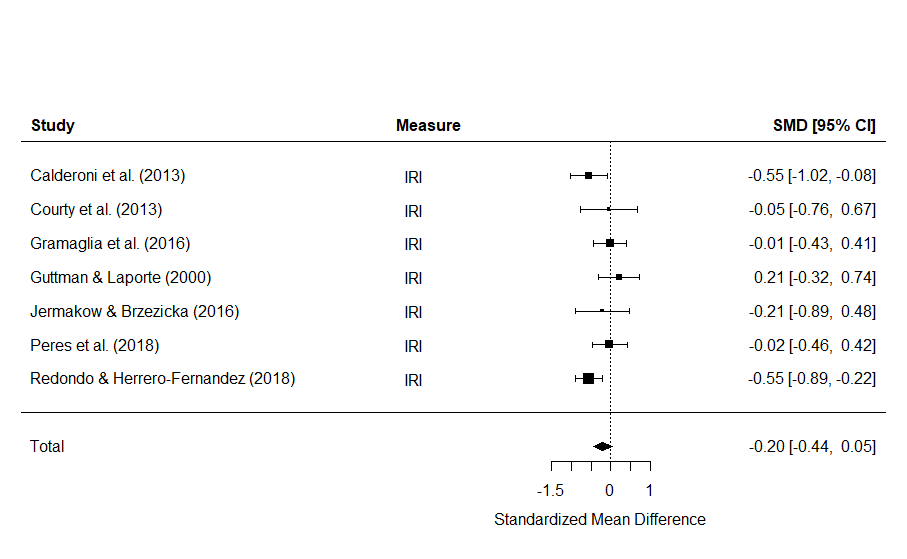


Figure D. Forest plot of standardized mean effect size for differences (SMD) between AN and HC on the perspective taking subscale of the IRI. Negative effect sizes indicate lower empathy scores in the AN group.


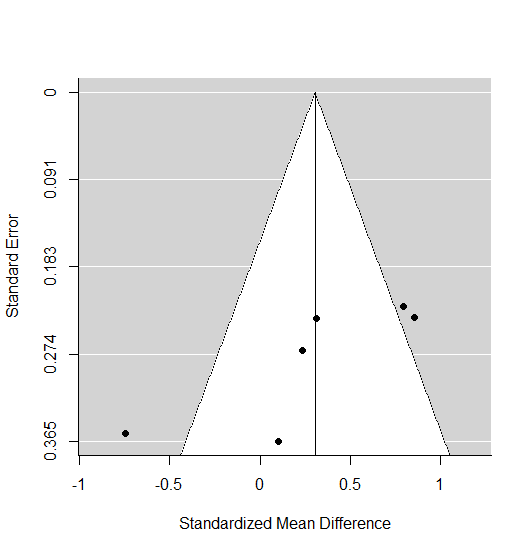


Figure E. Funnel plot of personal distress subscale scores to assess publication bias (p = 0.06)


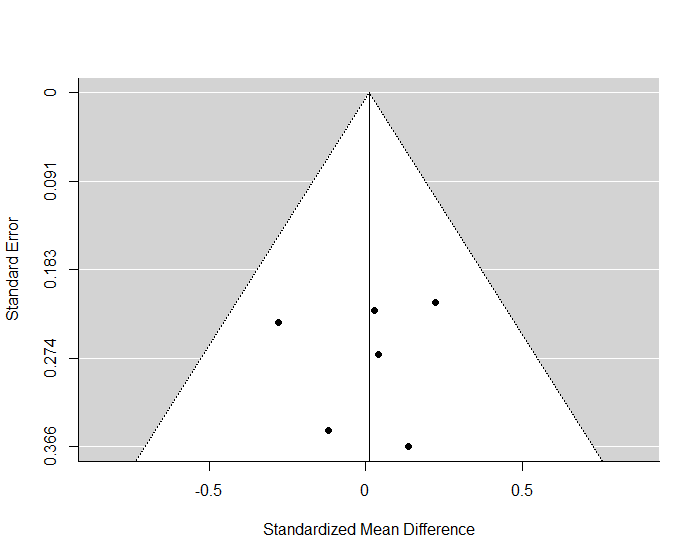


Figure F. Funnel plot of empathic concern subscale scores to assess publication bias (p = 1.00)


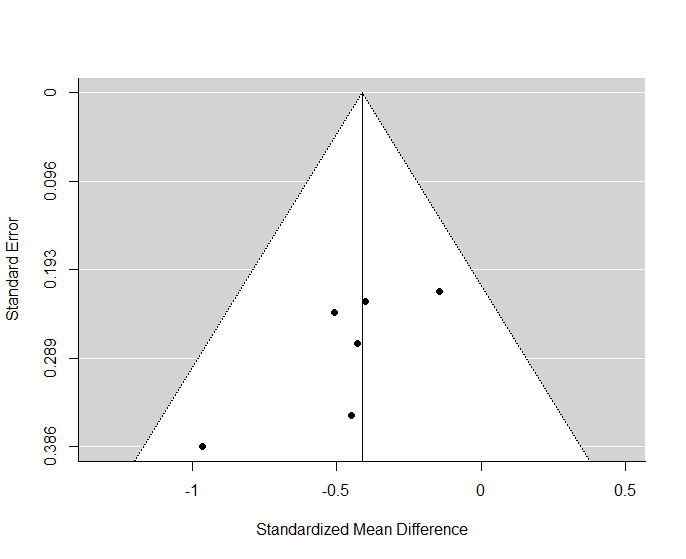


Figure G. Funnel plot of fantasy subscale scores to assess publication bias (p = 0.06)


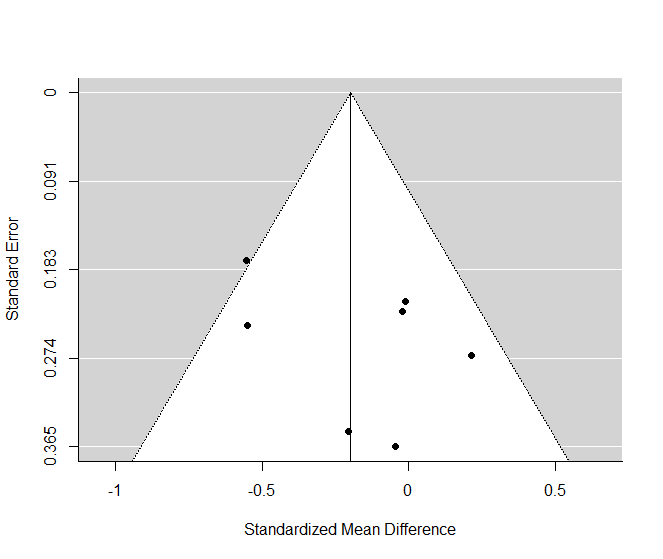


Figure H. Funnel plot of perspective taking subscale scores to assess publication bias (p = 0.77)
