# Supplementary material for: Cognitive and Affective Empathy in Eating Disorders: A Systematic Review and Meta-Analysis
Source: Front Psychiatry. 2019 Mar 4;10:102. doi: 10.3389/fpsyt.2019.00102 (PMC6410675; doi:10.3389/fpsyt.2019.00102)
Supplement: Supplementary file 2 [file Data_Sheet_2.docx]

| **Study** | **Clearly focused issue?** | **Appropriate method?** | **Case recruitment** | | | | **Control recruitment** | | | | **Exposure accurately measured** | | **Confounding factors accounted for in analysis? If so, what were the results?** | **Correlation between other variables assessed? If so, what was the result?** | **Precision of results** | **Results applicable?** | **Do results fit with available evidence?** | **Score (max 17)** |
| --- | --- | --- | --- | --- | --- | --- | --- | --- | --- | --- | --- | --- | --- | --- | --- | --- | --- | --- |
|  |  |  | **Cases defined?** | **Recruitment source given?** | **Exclusion/inclusion criteria?** | **Power calculation?** | **Controls defined?** | **Recruitment source given?** | **Exclusion/ inclusion criteria?** | **Matched to cases?** | **Empathy measure described?** | **Procedure?** |  |  |  |  |  |  |
| Adenzato et al. (2012) | Y | Y | Y | Y | Y | N | Y | N | Y | Sex, age, education | Y | Y | There were no differences in empathy between AN-R and AN-BP subtypes. | No correlation with BMI, age at illness onset, or duration of illness. Empathy correlated negatively with alexithymia, and positively with perceived social support. | Y | Y | Y | 15 |
| Aloi et al. (2017) | Y | Y | Y | Y | Y | N | Y | Y | Y | N | Y | N | Controlled for gender, depression, alexithymia on eating disorder psychopathology scores, group differences remained. | Alexithymia negatively correlated with empathy. | Y | Y | Y | 15 |
| Baron-Cohen et al. (2013) | Y | Y | Y | Y | N | N | Y | Y | N | Sex | Y | Y | Age entered as covariate in analyses, not reported whether there was an effect | No correlation between empathy and eating disorder psychopathology | N | Y | Y | 13 |
| Butler & Montgomery (2005) | Y | Y | Y | Y | N | N | Y | Y | N | Sex, age | Y | Y | Sub-group analyses of unmedicated patients only did not change the results. | Empathy did not correlate with performance on behavioural measures of impulsivity. | Y | Y | Y | 14 |
| Calderoni et al. (2013) | Y | Y | Y | Y | Y | N | Y | Y | Y | Sex, age | Y | Y | Significant differences in empathy sub-scales remained when ating disorder and general psychopathology scores were entered as covariates. | Cognitive empathy did not correlate with BMI, illness duration, or general psychopathology | Y | Y | Y | 16 |
| Courty et al. (2013) | Y | Y | Y | Y | Y | N | N | N | N | Sex, age, education | N | Y | N | Principal component analysis was used to analyse the correlation structure between autism traits, empathy, and alexithymia | Y | Y | Y | 11 |
| Duchesne et al. (2011) | Y | Y | Y | Y | Y | N | Y | N | Y | Age, education, income | Y | Y | N | N | Y | Y | Y | 13 |
| Feldman & Eysenck (1986) | Y | Y | Y | Y | N | N | N | N | N | Sex | N | Y | N | N | N | Y | N | 7 |
| Gramaglia et al. (2016) | Y | Y | Y | Y | Y | N | Y | Y | Y | Sex, age | Y | Y | N | N | Y | Y | Y | 14 |
| Guttman & Laporte (2000) | Y | Y | Y | Y | Y | N | Y | Y | Y | Sex | Y | Y | N | Empathy scores in AN and their parents were positively correlated, while there were no associations in HC and their parents. | N | Y | Y | 14 |
| Hambrook et al. (2008) | Y | Y | Y | Y | N | N | Y | Y | N | Sex | Y | Y | N | N | Y | Y | Y | 12 |
| Jermakow & Brzezicka (2016) | Y | Y | Y | N | N | N | N | Y | N | Sex | Y | Y | N | N | Y | Y | Y | 10 |
| Lule et al. (2014) | Y | Y | Y | Y | Y | N | Y | Y | Y | Sex, age, IQ, ethnicity | N | Y | N | N | Y | Y | Y | 13 |
| Morris et al. (2014) | Y | Y | Y | Y | Y | N | N | Y | Y | Sex | Y | Y | N | Total scores on the socio-emotional questionnaire were positively correlated with BMI and general functioning, and negatively correlated with depression. | Y | Y | Y | 14 |
| Nandrino et al. (2017) | Y | Y | Y | Y | Y | N | Y | Y | Y | Sex, age, education | Y | Y | N | No correlation between empathy and perception of personal space | Y | Y | N | 14 |
| Peres et al. (2018) | Y | Y | Y | Y | Y | N | Y | Y | Y | Sex | Y | Y | Anxiety did not explain differences between groups in personal distress or affective empathy | Personal distress and affective empathy positively correlated with anxiety but not depression | Y | Y | Y | 16 |
| Redondo & Herrero-Fernandez (2018) | Y | Y | Y | Y | N | N | N | Y | N | Sex | Y | Y | N | Empathy was positively correlated with reading the mind in the eyes task performance, and negatively correlated with alexithymia | N | Y | Y | 11 |
|  |  |  |  |  |  |  |  |  |  |  |  |  |  |  |  |  |  |  |
